# A Histologic Study on the Use of Tooth as a Graft Material in Oral Surgery: Analysis of 187 Samples

**DOI:** 10.3390/ma18112518

**Published:** 2025-05-27

**Authors:** Elio Minetti, Silvio Taschieri, Stefano Corbella

**Affiliations:** 1 Department of Biomedical, Surgical, and Dental Science, Università degli Studi di Milano, Viale Pisa 10, 20146 Milan, Italy; silvio.taschieri@unimi.it (S.T.); stefano.corbella@unimi.it (S.C.); 2 IRCCS Istituto Ortopedico Galeazzi, 20157 Milan, Italy; 3 Department of Oral Surgery, Institute of Dentistry, I. M. Sechenov First Moscow State Medical University, 119992 Moscow, Russia

**Keywords:** alveolar ridge reconstruction, autogenous, biomaterials, bone augmentation, bone grafting, bone substitutes, histological analysis, implantology, prospective

## Abstract

**Background**: To reconstruct and maintain hard tissues over time, it is necessary to follow effective protocols and use appropriate materials. The selection of the graft material and its properties can also affect the final outcomes. For this purpose, numerous graft materials have been suggested. Among the valuable alternatives to these biomaterials, interest in using teeth as graft material has grown in recent years. **Aim**: The aim of the study was to investigate the histomorphometric outcomes of using tooth-derived materials when used as a bone substitute. **Methods**: We evaluated the histological results of autologous demineralized tooth graft material prepared using a Tooth Transformer device. A total of 187 histological samples from 186 subjects (52% male and 48% female, with an average age of 56.30 ± 12.97 years) were analyzed. The analysis focused on the total bone volume (BV%), residual tooth material (residual graft, TT%), and vital bone (VB%). The differences between the presence and absence of the resorbable membrane were also analyzed. **Results**: The amount of VB was 36.28 ± 19.09%, the residual graft TT was 9.6 ± 10.76%, and 46.96 ± 13.85% was the total bone volume (BV). The presence of membrane increased the amount of BV% and reduced the time to produce bone. **Conclusions**: The procedure using demineralized autologous tooth-derived biomaterial may be a predictable method for producing new vital bone capable of supporting dental implant rehabilitation and the use of membrane allow better results.

## 1. Introduction

The greatest resorption of the alveolar process occurs within the first 12 months after the extraction. Dental implants must exhibit dimensional congruence between implant size and the available bone volume, as well as an appropriate distribution of functional load, to ensure long-term stability. The maintenance of alveolar bone volume is therefore a critical prerequisite to meet these biomechanical requirements. This implies that the soft tissues will follow the resorption of the bone tissue which is directly associated with the reduction in bone volume [1]. Bone resorption can make implant prosthetic rehabilitation impossible. Examining 1244 publications from PubMed and 106 from Cochrane Literature revealed that the average bone resorption of the alveolar bone after an extraction is 1.76 ± 2.03 mm vertically and 3.87 mm horizontally [2]. In an analysis using 3D tools and comparing the results, it was demonstrated that the resorption of post-extraction sockets is linked to the bone phenotype. The phenotype with a greater buccal bone thickness experiences less resorption compared to one with inherently reduced thickness [3]. Another literature review, evaluating the healing phase after the extraction of a dental element, measured a reduction in width ranging from 2.6 to 4.6 mm and a reduction in height ranging from 0.4 to 3.9 mm [4]. A literature review evaluated different alternatives for maintaining bone volume, highlighting numerous techniques proposed over time. To reduce bone resorption, especially in cases where implant placement is planned, numerous techniques have been tested to preserve bone volume [5]. An analysis conducted on 2085 patients showed that, compared to spontaneous healing, all biomaterials used (autogenous, allogenic, or xenogenic) significantly reduced both horizontal and vertical post-extraction bone resorption [6].

Different types of graft materials have been utilized and not all bone substitutes are suitable for every clinical application, and they must be selected based on their intended purpose. An ideal bone substitute should be safe, cost-effective, and fully biocompatible and not induce any foreign body reactions or adverse inflammatory responses; it should be sterile and supportive, or at least not interfere with the bone formation process during healing. The biomaterial should be fully resorbable to create space for bone growth [7,8].

A literature review with meta-analysis suggests that all biomaterials have shown positive results in terms of bone regeneration and volume preservation, and none appear to be superior to the others [9].

Xenograft materials seem to have the ability to maintain space, but since they do not resorb over time, they prevent bone tissue from growing during the healing process. Therefore, the placement of these biomaterials in pocket preservation may even slow healing.

As the material itself occupies the space, it impedes the growth of additional bone [10]. Since bone grows more slowly compared to soft tissues such as the epithelium, GBR (Guided Bone Regeneration) involves the use of a membrane that covers the area of the bone defect. This membrane serves to create a protected environment. Here, the cells that form bone, stimulated by growth factors, can transform into osteoblasts and produce new bone without being hindered by other cells, such as fibroblasts [11].

In the search for different materials, the use of teeth as an autogenous bone substitute has been rediscovered. As early as 1967, their use was suggested for reconstructing bone tissue due to their origin and complete biocompatibility [12].

Dentin is composed of 65% inorganic material, hydroxyapatite, with bidimensional crystals that are approximately 10 times denser than the hydroxyapatite found in bone and about 300 times less dense than the hydroxyapatite that makes up enamel [13].

The remaining 35% of dentin is made up of proteins. A total of 90% of these proteins are primarily type 1 collagen, with the remaining 10% consisting of non-collagenous proteins. These proteins are similar to or identical to those found in bone tissue. For this reason, dental tissue is very similar to bone tissue and could potentially be used as an autologous bone substitute biomaterial [14].

Autologous bone tissue has a high rate of resorption. The higher density of dentin, compared to bone tissue, allows for slower resorption, maintaining space in a way that is more consistent with regenerative techniques [15].

Various techniques and devices have been proposed and described for utilizing tooth material as a bone substitute in socket preservation, horizontal/vertical regeneration and sinus lift [16,17,18,19,20].

Numerous literature reviews have analyzed various procedures and outcomes achieved with this biomaterial of natural autologous origin [21,22,23,24,25,26,27,28,29,30,31,32,33]. A procedure, demonstrated in a recent medical device, can reliably and automatically fragment, decontaminate, and demineralize tooth material for grafting, making it suitable for use in the same individual when needed [19,20,21,22,23,24,25,26,27,28,29,30,31,32,33,34,35,36,37,38,39,40,41,42,43,44,45,46,47,48,49,50,51,52,53,54,55,56,57,58,59,60,61,62,63].

Several multicenter studies are made from the same research group to evaluate the clinical and histological performance of the material obtained from this procedure [34,35]. To our knowledge, studies of histomorphometric healing after using tooth-derived bone graft material under different conditions and by using different techniques (such as the application of a barrier membrane) are missing. For this reason, the aim of the present study (a retrospective, observational, non-comparative study) was to more thoroughly assess the histomorphometric outcomes of using tooth-derived autogenous bone substitute material in a large cohort of patients undergoing regeneration procedures with a particular emphasis on the changes in Vital Bone percentage (VB%) over time. This study was conceived due to a lack of research with large sample sizes—and therefore adequate statistical power—regarding the histological outcomes of regenerations performed using dental-derived materials.

## 2. Materials and Methods

Subjects were recruited from patients requiring tooth extraction or regeneration in the upper and lower maxillae between May 2022 and January 2024. The study was performed following the standard protocol for socket preservation or regeneration and implant placement without adopting any experimental procedure. All data were anonymized. The present study was carried out following the principles embodied in the Helsinki Declaration in its latest form [36]. On 21 March 2019, the University of Chieti Ethics Committee authorized the clinical study protocol on a human model registered under the number: 638—21/3/19.

### 2.1. Inclusion and Exclusion Criteria

The histomorphometric analysis was conducted on samples selected based on the following inclusion criteria:

INCLUSION: Subjects who underwent surgical intervention for tooth extraction and alveolar ridge preservation (ARP) using only tooth-derived bone substitute (Tooth Transformer^®^—Tooth Transformer srl, Milan, Italy). Subjects who received implant placement at the same site of socket preservation. Subjects with partial loss of buccal or palatal bone walls. Subjects without any systemic diseases or conditions that could impair bone metabolism.

EXCLUSION: Cases involving pregnant or lactating women were excluded. Cases with a follow-up period exceeding nine months and those where the dental graft material was mixed with other graft materials were excluded.

All subjects gave signed and informed consent.

### 2.2. Surgical Protocol

All surgical procedures were performed by experienced clinicians with over 10 years of expertise in oral surgery. The preparation of the tooth-derived substitute material followed a standardized protocol. Initially, the extracted tooth was meticulously cleaned to remove any residual calculus and thoroughly polished using a diamond drill (ref. 6855—Dentsply Maillefer, Ballaigues, Switzerland) under abundant saline irrigation. This step ensured the complete removal of any root canal filling material from the selected tooth. Next, the tooth was sectioned into small fragments, which were then processed using the Tooth Transformer^®^ device (Tooth Transformer srl), following the protocols established in previously published studies [19]. In most cases, the entire tooth was utilized. However, for teeth that had undergone endodontic treatment or served as prosthetic supports, enamel was typically absent, as it had been removed during earlier preparatory steps. Additionally, most of the cementum was eliminated during the cleaning process, leaving dentin as the primary component of the tooth-derived material. After 25 min of processing, the material was ready for implantation into the recipient site. This site had been prepared by creating cortical perforations using a 1.5–2 mm spiral drill to facilitate and enhance the bone healing process [37]. The defects or cavities, whether related to socket preservation or ARP, were cleaned using ultrasonic instruments after tissue elevation. After being cleaned with a piezoelectric instrument and a sharp tip, the cavities underwent cortical perforations to promote vascularization and enhance the regenerative potential of the graft. This procedure was performed prior to the placement of the dental-derived biomaterial, in order to optimize integration and bone regeneration within the defect site. The material produced by the Tooth Transformer was then condensed. One hundred forty-eight cases were covered with an OsseoGuard^®^ collagen membrane (Collagen Matrix, Oakland, NJ, USA) and 39 cases were closed without membrane. The tissues were subsequently closed by primary intention using 5.0 Ethicon sutures (Johnson & Johnson, Brunswick, NJ, USA). After grafting, the defect was covered with a resorbable membrane (Osseo Guard—Zimmer Biomet, Warsaw, IN, USA). Dental implants were subsequently placed in the grafted area following a healing period of 3 to 9 months. Implant site preparation was performed using a 3 mm trephine bur (Meisinger, Centennial, CO, USA) under abundant saline irrigation, occasionally in combination with other specialized drills, following standard protocols. Bone tissue samples were harvested using a trephine bur and immediately immersed in formalin. The implants were inserted with varying torque values, and the post-implant healing period was no less than 3 months. In the postoperative phase, all patients received a six-day course of antibiotic prophylaxis with amoxicillin, as well as analgesic and anti-inflammatory therapy with naproxen sodium, in accordance with standard clinical protocols for pain and infection management.

### 2.3. Histological Technique

The samples were analyzed within 7 days. The specimens were decalcified, embedded in paraffin, and sectioned. Samples were fixed in 10% neutral buffered formalin (containing 10 mL of 37% formaldehyde solution, 0.8 g NaCl, 0.4 g potassium phosphate monobasic, 0.65 g potassium phosphate dibasic, and 90 mL distilled water) for 7 days. Decalcification was performed using disodium EDTA at pH 7 until complete decalcification, with the endpoint determined by physical examination. Subsequently, the specimens were dehydrated in ethanol solutions of increasing concentrations (70% to 100%), cleared with xylene, and embedded in paraffin. All chemicals were sourced from Carlo Erba Reagents (Cornaredo, Italy). Paraffin sections were prepared using a Leica RM2245 rotary microtome and placed on Superfrost microscope slides, which were mounted with Biomount HM (Bio-Optica, Milan, Italy). Histological images obtained via a transmitted light microscope (Olympus, Tokyo, Japan) were digitized using a digital camera and analyzed with IAS 2000 image analysis software (QEA). Histomorphometric analysis distinguished the following parameters: BV% (Bone Volume): the percentage of mineralized tissue, excluding medullary tissue. TT% (Tooth Tissue): the percentage of volume occupied by the remaining graft material, specifically dentin. VB% (Vital Bone): the percentage of vital bone, excluding medullary tissue. The BV% was calculated as the sum of TT% and VB%. Measurements for each section were performed using the ImageJ software 1.52 version. The thickness of the sections was 4 μm. The cores were sectioned, and three sections corresponding to the first, second, and third thirds were selected. Quantification was performed by two independent observers. The longitudinal sections were stained with hematoxylin and eosin and examined at 200× magnification.

### 2.4. Statistical Methods

Descriptive statistics for continuous variables were reported as mean, median, and standard deviation. For categorical variables, frequencies were calculated. The normality of variable distributions was assessed using the Shapiro–Wilk test. Differences based on sex, location (maxilla vs. mandible), type of bone defect, and healing time were analyzed using ANOVA and Student’s *t*-test for variables with normal distributions, including BV%, TT%, and VB%. Linear regression analysis was conducted to evaluate the impact of baseline factors (sex, healing time, location, and defect type) on the histomorphometric parameters BV%, TT%, and VB%.

## 3. Results

**Figure 1:**
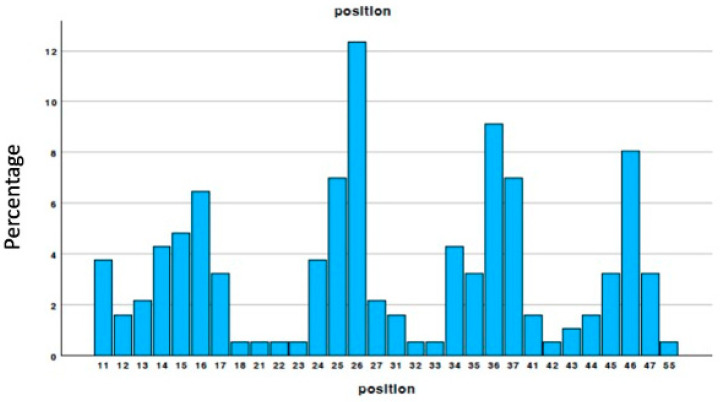
Distribution of the samples (tooth position).

We analyzed histologies collected from 118 males and 110 females, with an average age of 56.30 ± 12.968. Forty-one histologies were excluded from the study as they were produced by mixing the treated tooth with market biomaterial. Thus, the data that met the inclusion and exclusion criteria resulted in 187 cases, of which 90 were female and 97 were male. Figure 1 shows the distribution of the samples by biopsy site. It is noteworthy that the highest number of samples were taken from the areas around the first molars, consistent with the common literature on the most frequent zones of regeneration.

**Figure 2:**
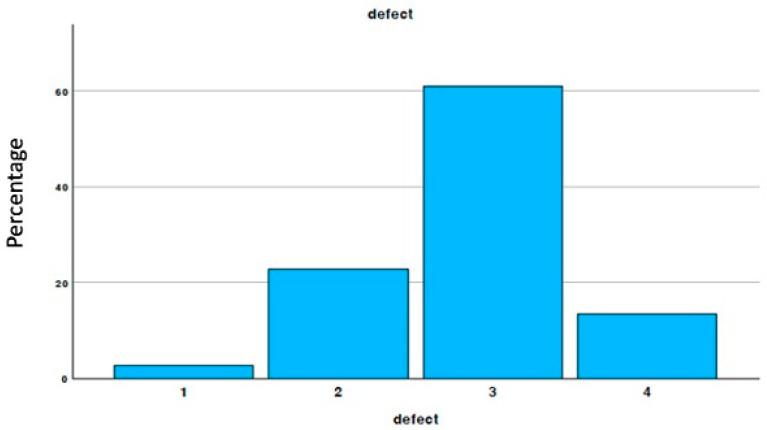
Percentage of defect type.

The defects were classified as one-wall defects, two-wall defects, three-wall defects, and four-wall defects, as shown in Figure 2. Of these, 50% were three-wall defects.

Table 1 shows the residual dentin graft in relation to the presence or absence of a membrane covering the defect during the surgical phase. The healing time and the presence of the membrane was significantly correlated (positively) to the amount of vital bone (*p* = 0.05). In Table 2 data about sites treated with or without membranes are presented. There was a statistically significant difference between the two groups in terms of proportion of vital bone, favoring the “membrane” group, thus allowing us to hypothesize that the presence of a barrier membrane may enhance the graft resorption rate and, conversely, the new bone formation.

These data are also presented in Table 1, where the average values of vital bone with and without the membrane are indicated. The amount of VB was 36.28 ± 19.09%, the amount of residual graft TT was 9.6 ± 10.76%, and 46.96 ± 13.85% was the amount of total bone volume BV (Table 3). A 20% variation in bone volume achieved in a shorter time allows for the placement of smaller implants, increasing the likelihood of successful osseointegration, reducing waiting time, and ultimately improving patient comfort and surgical simplicity for the clinician

Table 1 presents the residual tooth (dentin graft) in different follow-up periods. As it is reported, the median residual tooth graft decreased particularly between the three-month and subsequent timeframes, both in the membrane group and in the group in which membrane was not used. Comparing the trends in the two groups, the median value was significantly lower in the membrane group as compared to other cases, and this can be interpreted (with caution since the present was not a randomized clinical trial) as the result of the membrane’s ability to allow a higher graft resorption rate even in the short term. The amount of vital bone over time is shown as a consequence. The proportion of vital bone depends on the possibility of substituting the tooth graft during the healing period and follows an inverse pattern. The more the tooth graft is substituted, the more vital bone is present, so, for the same reasons, placing a membrane to cover the defect may allow a higher and earlier graft substitution and this is particularly evident in the first time frames, between 3 and 5 months.

**Figure 3:**
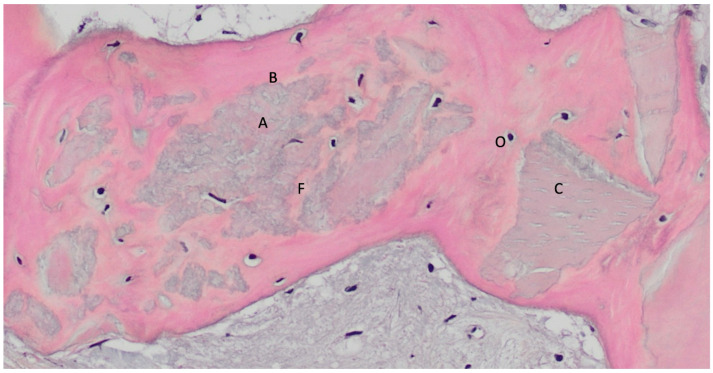
Hybrid bone–dentin tissue. This image shows a region where dentin (A) and bone (B) form a hybrid tissue, with the boundary between the two not easily distinguishable. The dentin still retains its dentinal tubules, visible in longitudinal section (C). The area occupied by the bone tissue is indicated by the presence of osteocytes (O), but the transition between the two tissue types is indistinct (F). Decalcified section, magnification 200×, stain: hematoxylin and eosin. (Histology performed by P. Savadori).

**Figure 4:**
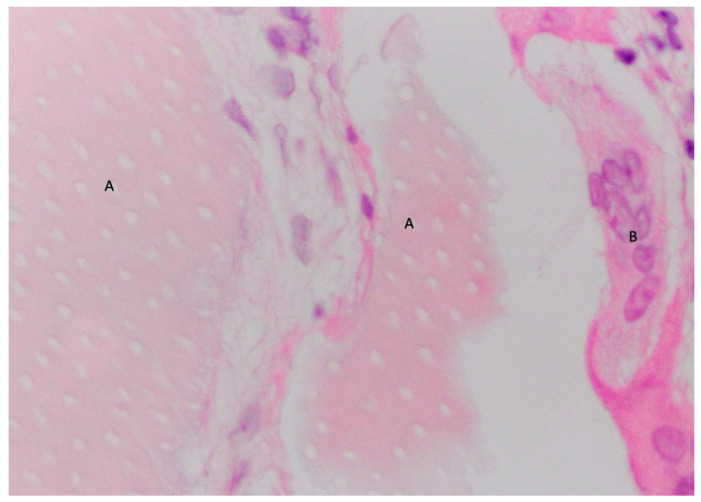
Two dentin granules still contain dentinal tubules, visible in longitudinal section (A) showing a characteristic sign of resorption by osteoclasts (B). Decalcified section, magnification 300×, stain: hematoxylin and eosin. (Histology performed by P. Savadori).

**Figure 5:**
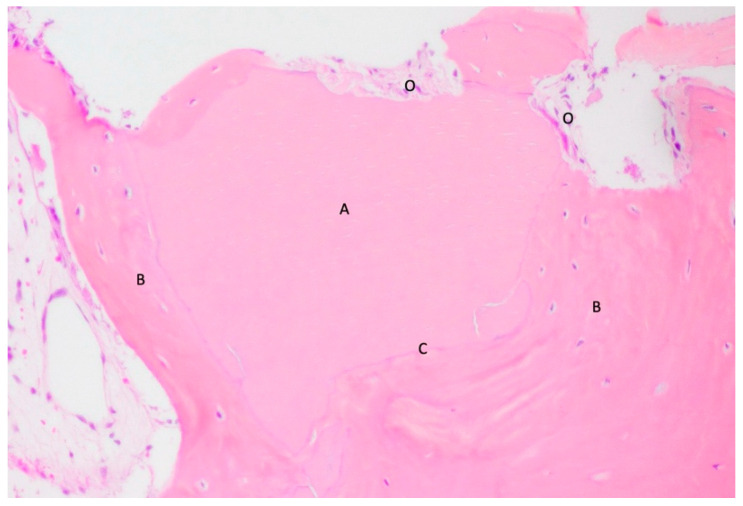
Dentin granule (A) completely buried in the bone (B) during the resorption phase by osteoclasts (O). The purple collagen line, known as the cement line (C). Decalcified section, magnification 200×, stain: hematoxylin and eosin. (Histology performed by P. Savadori).

**Figure 6:**
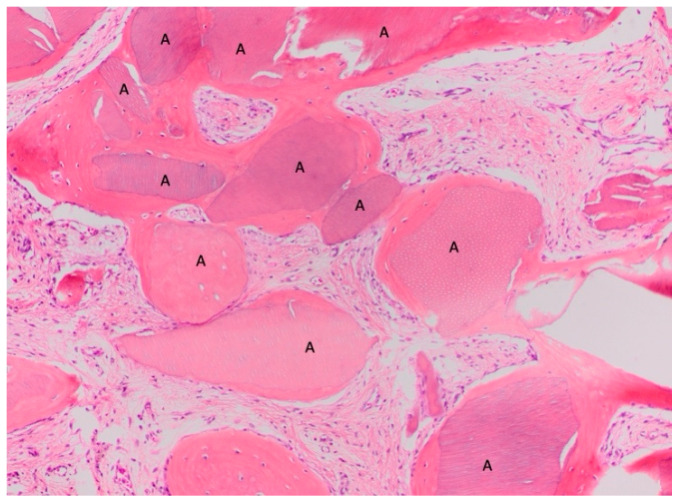
Dentin granules (A) are completely embedded in the bone, with the purple collagen line known as the cement line. Is it interesting to note that the color of the dentin and bone is very similar. Both tissues absorb the stain in the same way. Magnification 200×, stain: hematoxylin and eosin. (Histology performed by P. Savadori).

**Figure 7:**
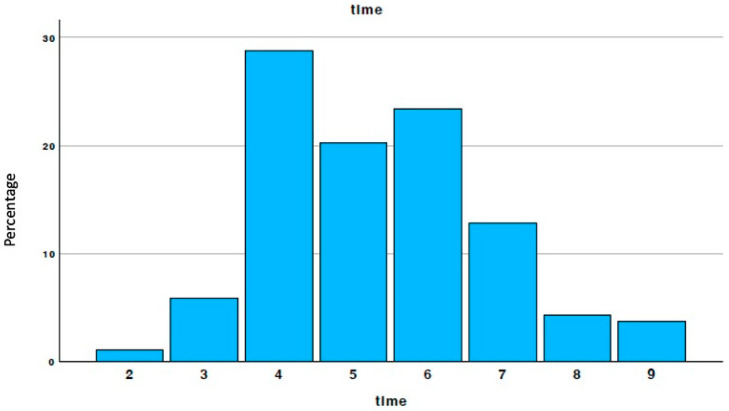
Timing distribution of the biopsies.

In the analyzed histologies, different stages of dentin granule resorption were observed (Figure 3), including the presence of osteoclasts actively resorbing dentin granules (Figure 4 and Figure 5), dentin granules completely surrounded by newly formed bone with a cement line (Figure 6), and complete histological images showing residual dentin. The mean values of the histomorphometric analyses are presented in Figure 7.

The biopsies were performed between three and nine months after the regenerative surgical phase, during implant placement. The timing of the biopsies was determined by the specific needs of the patients and the dentists as shown in Figure 7.

## 4. Discussion

Numerous studies have been conducted considering a large number of clinical trials and various materials [38,39]. The performance of the xenogenic materials was evaluated through systematic reviews and meta-analyses. Regarding the amount of residual graft material, the lowest percentages were observed with allografts (12.4–21.11%), while procedures using xenogenic or alloplastic materials showed residual amounts of 37.14% and 37.23%, respectively, at seven months [40]. It has been shown that, when sealed with a collagen membrane, from a clinical perspective, this type of graft can reduce the three-dimensional shrinkage of the bony crest. However, from a histological standpoint, these materials, due to their manufacturing process, may exhibit incomplete resorption even over the long term. The evolution of medicine increasingly emphasizes personalized, minimally invasive therapies to reduce the surgical impact on patients [41,42]. Factors such as the preservation and reuse of tissues or the creation of “biomimetic” materials should be considered important in reducing the impact of surgical procedures on patients. Promising materials being used as alternatives to common synthetic or animal-derived biomaterials for bone regenerative procedures, are attracting much attention, particularly teeth and tooth derivatives through specific treatments [43,44].

Using autologous teeth, rather than discarding them, offers significant cost savings for both the patient and the clinician, compared to the use of expensive heterologous or synthetic bone substitutes. This approach is likely to be well-received by patients, particularly because it utilizes a part of their own body that has been extracted. The graft is sourced from the patient’s extracted tooth and is processed using a newly developed device that shreds and fully decontaminates dental materials, converting it into grafting material suitable for addressing various bone defects in oral surgery procedures [45].

Following the procedure—which requires 1 to 5 min for cleaning and cutting the tooth (depending on its condition) and 25 min for processing—it is possible to obtain 0.5–3 g of material, depending on the size of the tooth. The key advantages of using this material are as follows: it is entirely autogenous; it eliminates the need for an additional surgical site to harvest bone graft; and the structure and composition of dentin closely resemble those of bone [46]. After the demineralization process, the proteins contained in the material, including BMP-2, endow it with osteoinductive properties in addition to the osteoconductive characteristics provided by the porous three-dimensional matrix [15,47,48].

Regarding the presence of BMP-2 after treatment with Tooth Transformer, recent research made from University of Foggia have demonstrated the retention of BMP-2 proteins despite the treatment involving acids [49].

The extracted tooth can be stored for an extended period before surgery (TH Schmidt-Schultz) [50,51].

The ability to store teeth allows for the use of teeth extracted years ago, as well as deciduous teeth [52].

In our previous publication, “Tooth graft material: Histological study”, we analyzed 101 samples [53]. Increasing the number of tests performed, this second prospective study of the histological and histomorphometric analyses of dentin treated with a tooth transformer was conducted with a larger sample size. It is interesting to note that the histomorphometric values from the present study and the one conducted by the same group in 2022, which involved a total of 288 histological samples, are remarkably similar in terms of both vital bone (VB) quantity and tooth graft residue (TT) [53]. The greater amount of vital bone allows for improved implant stability and enhances the chances of successful osseointegration.

**Figure 8:**
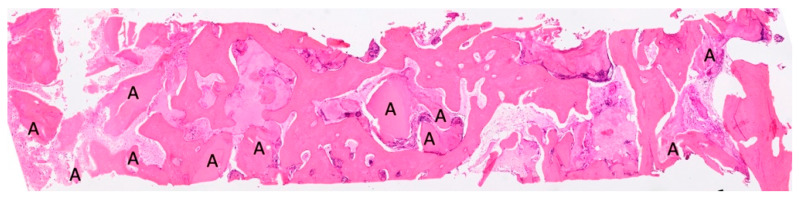
In this histological section, dentin granules (A) are visible, distributed throughout the entire sample, indicating that the tissue was taken from the center of a regenerated area. This suggests that the resorbed granules have transformed into bone. BV 53,659%, VB 45,420%, residual graft TT 8239%. Magnification: 70×, stain: hematoxylin and eosin. (Histology performed by P. Savadori).

**Figure 9:**
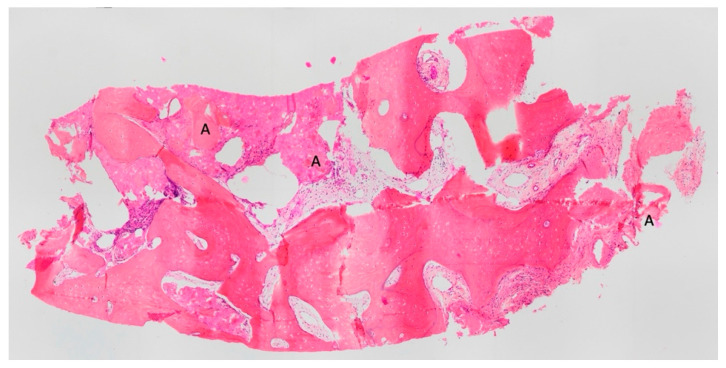
In this histological section, dentin granules (A) are visible, distributed throughout the entire sample, indicating that the tissue was taken from the center of a regenerated area and that the resorbed granules have transformed into bone. BV 57,791, VB 56,978%, residual graft TT 0.813%. Magnification 70×, stain: hematoxylin and eosin. (Histology performed by P. Savadori).

All the biopsies were performed in cavities completely filled with dental graft. In Figure 8 and Figure 9, the dentin granules are visible, distributed throughout the entire histological section, indicating that the histological sample was taken from the center of a regenerated area and that the resorbed granules have been transformed into bone.

Regenerative biomaterials are designed to provide mechanical support to bone tissue while ensuring biocompatibility and resorption that allies with bone remodeling processes. To achieve this, pore size and density are crucial factors [54].

The bone is naturally porous, with pore sizes ranging from 1 to 100 μm (55–57). Dentin contains dental tubules. After demineralization treatment, these tubules become enlarged and closely resemble the porosities of bone tissue, as demonstrated by Tanoue [58]. The newly formed bone matrix can deeply infiltrate the non-resorbed dentin granules, a phenomenon attributed to the body’s recognition of dentin as being analogous to its own bone tissue [53].

This study analyzes the regenerated volumes or their progression over time, but further focuses on the biopsies of the residual volume.

A three-dimensional analysis would be valuable for a comprehensive comparison with other graft materials. The amount of new bone and residual graft closely resembles that of autologous bone tissue [59].

Bone density following a regenerative procedure is influenced by several factors, including healing time, patient age and gender, the specific jaw (maxilla or mandible), and the location in the mouth (posterior or anterior) [60].

**Figure 10:**
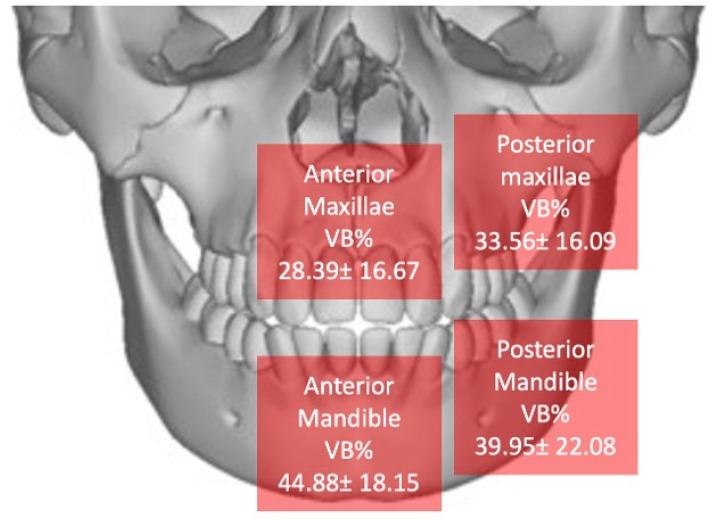
Distribution of new bone across different areas.

According to the previous article [53], the newly regenerated tissue using the tooth as a graft undergoes a form of homologation with the receiving tissue, particularly after the stimulation from granule resorption diminishes. This process, referred to as proximity homologation, is driven by the natural mechanisms of bone metabolism. The tooth functions similarly to autologous bone, with the resorptive pattern tending to align with the natural anatomy of the original ridge. This effect implies that the bone tissue formed during regeneration is similar in density and NB% to native bone tissue. Therefore, bone density will be lower in maxillary areas and higher in mandibular areas. We processed the data and assessed this aspect in Figure 10. Indeed, as previously demonstrated in another study, using dentin can achieve this result. The image shows the average VB% for each area, confirming previously expressed impressions [61].

The non-resorbed granules serve as a foundation for the deposition of a new bone matrix, which is generated by osteoblasts around the granules (Figure 6).

An interesting evaluation of the obtained data is presented in Table 3. It shows that, under equivalent time conditions, the amount of new bone produced is lower when a membrane is not used. Additionally, the graph highlights that the absence of coverage with a resorbable membrane results in a temporal delay in bone production.

Similar results with the same materials were found in this previous clinical–histological study [62].

## 5. Limitations, Clinical Implications and Future Research

A limitation of this study, despite the large sample size, is the lack of a comparative study. Future research studies on the same topic that are structured in randomized controlled clinical trials may help to highlight the advantages in using this material over the others, if any. Moreover, future RCTs will help to compare clinical efficacy/effectiveness and to also explore patient-reported outcomes. Further multicentric clinical randomized study design with longer follow-up, larger sample sizes, and extended observation periods should be conducted, comparing the results to a gold standard, to effectively evaluate and understand the true impact of demineralized tooth graft materials on bone regeneration in oral and maxillofacial procedures. The clinical implications of these results, with a higher percentage of vital bone, suggest better conditions for implant placement, as this implies more effective osseointegration, as well as lower costs and greater patient acceptance.

## 6. Conclusions

Within the limitations of the present study, natural teeth may be considered a viable source of bone substitute material. From a clinical point of view, the relatively high resorption rate and substitution with vital bone is a significant outcome that may support the use of tooth as a graft material in the described type of intervention. Ideally, we should admit that the higher the percentage of vital bone we have, the better the conditions for placing dental implants, since it implies a more effective osseointegration. The use of the membrane and the values of newly formed bone resulting from the use of this natural biomaterial are clinically relevant.

Further multicentric clinical randomized study design with longer follow-up, larger sample sizes, and extended observation periods should be conducted, comparing the results to a gold standard, to effectively evaluate and understand the true impact of demineralized tooth graft materials on bone regeneration in oral and maxillofacial procedures.

## Figures and Tables

**Table 1:** Mean values ± standard deviations of histologic parameters over time, comparing membrane vs. no membrane. In bold, when the difference is statistically significant. The two-month timeframe was not presented because of the numerosity of the sample at that time.

		3 Months	4 Months	5 Months	6 Months	7 Months	8 Months	9 Months
Vital bone %	Membrane	64.21 ± 23.04	**80.05 ± 25.08**	**83.19 ± 20.09**	77.92 ± 23.30	79.38 ± 22.48	81.23 ± 26.65	75.32 ± 20.09
	No membrane	29.38 ± 43.51	**54.98 ± 41.98**	**61.07 ± 32.60**	68.04 ± 27.55	79.52 ± 17.63	77.13 ± 0.00 [n = 1]	55.27 ± 0.00 [n = 1]
Tooth graft %	Membrane	35.79 ± 23.05	19.16 ± 25.07	15.82 ± 19.05	21.88 ± 23.43	20.47 ± 22.55	18.49 ± 26.43	17.95 ± 16.81
	No membrane	59.52 ± 36.19	26.24 ± 26.04	26.43 ± 23.83	31.96 ± 27.55	20.74 ± 18.02	22.87 ± 0.00 [n = 1]	44.73 ± 0.00 [n = 1]

**Table 2:** The analyses showed that the presence of the membrane alters the percentages of vital bone and tooth graft. There was a statistically significant difference between the two groups in terms of proportion of vital bone, favoring the “membrane” group, thus allowing us to hypothesize that the presence of a barrier membrane may enhance the graft resorption rate and, conversely, the new bone formation.

	No Membrane	Membrane Yes	*p* Value
% tooth graft	33.15 ± 27.18% [CI95%: 24.72–41.58]	19.20 ± 22.57% [CI95%: 15.77–22.63]	Statistically non significant *p* = 0.073
% vital bone	57.34 ± 34.89% [CI95%: 46.54–68.14]	78.95 ± 23.79% [CI95%: 74.34–82.56]	Statistically significant *p* < 0.001

**Table 3:** Histomorphometric analysis data from 187 samples analyzed.

	Numerosity	Minimum	Maximum	Mean	Dev.st.
Bone Volume	187	11.120	90.416	46.96537	13.855495
Tooth Graft	187	0.000	56.010	9.60344	10.768212
Vital Bone	187	0.000	90.416	36.28229	19.095735
Xenograft	0				

## Data Availability

The original contributions presented in this study are included in the article. Further inquiries can be directed to the corresponding author.
